# Thermomechanical Treatment-Enabled Short-Circuit Diffusion Enhances Molten-Carbonate Corrosion Resistance of an Alumina-Forming Austenitic Alloy

**DOI:** 10.3390/ma19061206

**Published:** 2026-03-19

**Authors:** Haocheng Jiang, Haicun Yu, Yuehong Zheng, Faqi Zhan, Peiqing La

**Affiliations:** State Key Laboratory of Advanced Processing and Recycling of Non-Ferrous Metals, School of Materials Science and Engineering, Lanzhou University of Technology, Lanzhou 730050, China

**Keywords:** molten salt corrosion, alumina-forming austenitic alloy, thermomechanical treatment, short-circuit diffusion, grain boundary engineering, oxidation kinetics

## Abstract

Developing stable alumina-based scales is critical for alumina-forming austenitic (AFA) alloys exposed to highly basic molten carbonates. However, the inherently sluggish diffusion of Al in austenite often limits the establishment of continuous protective layers. Herein, a thermomechanical treatment (TMT) strategy is proposed to enhance short-circuit diffusion pathways and promote selective Al oxidation in a Li–Na–K carbonate melt at 700 °C. After 90% cold rolling, annealing at 800 °C and 1000 °C generated two distinct microstructural states characterized by different grain boundary types, dislocation densities, and NiAl precipitate populations. The 800 °C-annealed alloy exhibits a significantly lower steady-state corrosion rate (~62 μm/yr) compared with the coarse-grained 1000 °C counterpart. EBSD and TEM analyses reveal that ultrafine grains, abundant low-angle boundaries, and finely dispersed NiAl precipitates provide efficient fast-diffusion channels and local Al reservoirs, enabling rapid formation of a continuous LiAlO_2_/Al_2_O_3_ inner layer. In contrast, insufficient Al flux in the 1000 °C microstructure results in extensive internal oxidation and growth of a thick, non-protective LiFeO_2_/NiO scale. These findings demonstrate that controlling the defect and grain-boundary structure via TMT is an effective route to overcome Al diffusion limitations and improve the molten-carbonate corrosion resistance of AFA alloys.

## 1. Introduction

The drive for higher thermal efficiency in next-generation concentrated solar power (CSP) systems is pushing the operating temperature of molten-salt heat-transfer media beyond 650 °C [1,2,3,4]. Ternary Li–Na–K carbonate salts are promising candidates in this temperature regime, but their high corrosivity poses a severe challenge to structural materials [5,6]. Ni-based alloys provide excellent corrosion resistance, yet their high cost limits large-scale deployment [7]. Conventional austenitic stainless steels rely on Cr_2_O_3_-based scales; however, chromia dissolves readily in basic molten carbonates through the formation of soluble chromates [8,9]. As a result, alumina-forming austenitic (AFA) alloys have emerged as a more cost-effective alternative [7]. Owing to the superior thermodynamic stability of α-Al_2_O_3_ and LiAlO_2_ in molten carbonate melts, Al-containing alloys such as OC-4 and HR224 have demonstrated improved corrosion resistance compared with Cr-forming steels [10,11,12,13,14,15].

To fully appreciate the severity of material degradation, the unique chemistry of molten carbonates must be considered within the Lux–Flood acid–base framework. In this system, melt basicity is governed by the activity of oxide ions, a(O^2−^). The dissociation of carbonate ions establishes a dynamic equilibrium that depends on the partial pressure of CO_2_, pCO_2_. Under open-air conditions, where pCO_2_ is relatively low, the equilibrium shifts toward the generation of free oxide ions, resulting in a highly basic melt [16]. This strong basicity represents the “Achilles’ heel” of chromia-forming alloys. According to solubility maps of metal oxides in molten carbonates, Cr_2_O_3_ exhibits amphoteric behavior but becomes highly soluble in the basic regimes, dissolving as chromate ions (${CrO}_{4}^{2-}$) [17]. This dissolution undermines the self-healing capability of standard stainless steels such as SS310 and SS316. In contrast, α-Al_2_O_3_ and alkali aluminates (e.g., LiAlO_2_) display much broader stability windows and significantly lower solubility limits in basic melts [18]. Therefore, shifting from chromia- to alumina-based protection is not merely beneficial but essential for ensuring the survivability of CSP components operating above 650 °C.

However, the protective efficacy of AFA alloys is fundamentally governed by the diffusion kinetics of aluminum. To form an exclusive external alumina scale, the outward flux of Al must exceed a critical threshold to prevent internal oxidation. This requirement is particularly challenging in austenitic matrices, where Al diffusion is inherently sluggish. Microstructural engineering via thermomechanical treatment (TMT) offers a pathway to overcome this kinetic barrier. While some studies on 316L and Inconel 601 have suggested that high defect densities produced by cold working can accelerate corrosion by providing inward diffusion paths for oxidants [19,20], defects such as grain boundaries and dislocations also serve as short-circuit diffusion paths for solute elements including Al and Cr [21]. This dual role introduces a key scientific question: Can a tailored TMT process introduce sufficient fast-diffusion channels to accelerate protective scale formation without compromising the alloy’s structural integrity in molten carbonates?

In this work, we propose a microstructure-oriented strategy to enhance the corrosion resistance of an AFA alloy. Cold-rolled specimens were annealed at two distinct temperatures (800 °C vs. 1000 °C) to selectively tailor the grain boundary character and dislocation density. The resulting microstructures were then exposed to ternary Li–Na–K carbonates at 700 °C. By correlating corrosion kinetics with EBSD and TEM analyses, we elucidate how short-circuit diffusion induced by grain refinement and lattice defects facilitates rapid formation of a continuous LiAlO_2_-based protective layer. These results provide metallurgical guidelines for designing durable AFA alloys for next-generation high-temperature thermal energy storage systems.

## 2. Experimental and Methods

### 2.1. Material Preparation

The starting material was a 4 mm thick hot-rolled AFA alloy plate. The chemical composition is listed in Table 1. The alloy design is based on conventional 800H heat-resistant steel, modified by a 3.33 wt.% Al addition to promote alumina scale formation, while adjusting the Ni and Cr equivalents to maintain a fully austenitic matrix. The plate was cold-rolled with a 90% thickness reduction to a final thickness of 0.4 mm using a two-high rolling mill. Subsequently, the cold-rolled sheets were annealed at 800 °C and 1000 °C for 60 min in an ambient-air furnace, followed by air cooling. To eliminate the oxide scale formed during annealing, the surfaces of all heat-treated specimens were ground with SiC papers up to 2000 grit to expose fresh metal prior to corrosion testing. These two temperatures were selected because 1000 °C corresponds to the full recrystallization regime of this alloy (producing a stress-free, coarse-grained matrix), whereas 800 °C lies in the partial recovery/recrystallization regime, deliberately retaining a high density of dislocations and subgrain boundaries.

### 2.2. Molten Salt Corrosion Tests

Specimen preparation: Rectangular specimens (12 × 12 × 0.4 mm^3^) were cut from the annealed sheets by wire electrical discharge machining (WEDM). The surfaces were ground and then ultrasonically cleaned in ethanol. Dimensions were measured with a digital micrometer to calculate the initial surface area (S_0_).

Salt and crucible: A eutectic ternary carbonate mixture (Li_2_CO_3_–Na_2_CO_3_–K_2_CO_3_ at 32.1:33.4:34.5 wt.%) was used. Reagent-grade powders were mixed and pre-dried at 120 °C for 24 h to remove adsorbed moisture. High-purity alumina crucibles were used to contain the melt. A sufficient amount of salt was added to fully immerse the specimens and to minimize changes in melt composition during exposure.

Immersion test: The corrosion tests were performed in a box furnace at 700 °C under a static air atmosphere, simulating open-system conditions. Specimens were fully immersed in the molten salt. Exposures were interrupted at intervals of 60, 120, 180, 240, and 500 h. After each interval, crucibles were air-cooled, and the solidified salt was dissolved in warm distilled water to recover the specimens.

**Figure 1:**
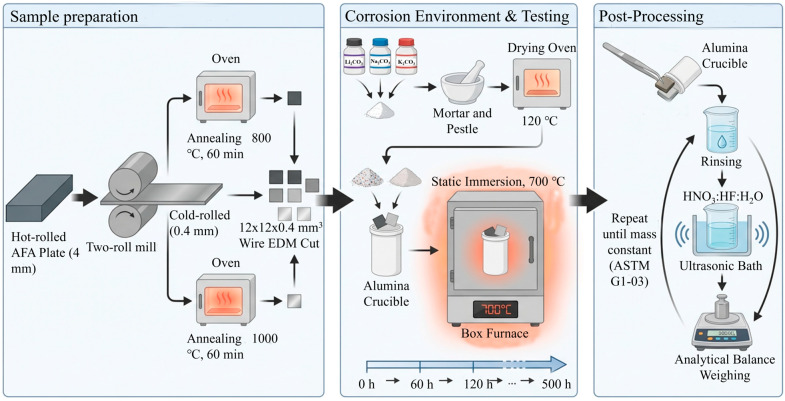
Schematic diagram of the experimental procedure for specimen preparation and molten salt corrosion testing.

Descaling process: To evaluate metal loss, corrosion products were removed chemically following the ASTM G1-03 standard [22]. Specimens were ultrasonically cleaned in a HNO_3_:HF:H_2_O solution (5:1:1 vol.) at room temperature. A repetitive cleaning–weighing cycle was employed to ensure complete removal of oxides while minimizing any attack on the substrate. For each condition and exposure time, three specimens were tested in parallel, and the reported values represent the average of these three measurements. Figure 1 illustrates the detailed process flow for specimen preparation and subsequent molten salt corrosion testing.

### 2.3. Corrosion Rate Calculation

The corrosion resistance was evaluated based on the mass loss per unit area. The corrosion rate (R_corr_, μm/year) was calculated using Equation (1):

(1) $$R_{\mathrm{corr}}\;=\;\frac{8.76\;\times \;10^{7\;}\times \;\Delta m}{S_{0}\cdot t\cdot \rho }$$

where Δm represents the mass loss (m_0_ − m_t_, in g), S_0_ is the initial surface area (cm^2^), t is the exposure time (h), and ρ is the alloy density (7.8 g/cm^3^).

### 2.4. Microstructural Characterization

Phase identification of the corrosion products was performed by X-ray diffraction (XRD, DX-2800, Rigaku Corporation, Tokyo, Japan) using Cu Kα radiation (λ = 1.5406 Å) in the 2θ range of 10–120°. Surface and cross-sectional morphologies were examined using field-emission scanning electron microscopy (FE-SEM, Quanta 5000, FEI Company, Hillsboro, OR, USA) equipped with energy-dispersive spectroscopy (EDS). For cross-sectional analysis, corroded specimens were cold-mounted in epoxy resin to preserve the integrity of the oxide scale. Crystallographic orientations and grain boundary characteristics of the as-annealed specimens were analyzed by electron backscatter diffraction (EBSD, Symmetry, Oxford Instruments, Abingdon, UK) with a step size of 30 nm; the data were processed using Channel 5 (version 5.12) software (Oxford Instruments, Abingdon, UK). Transmission electron microscopy (TEM, JEM-F200, JEOL Ltd., Tokyo, Japan, 200 kV) was employed on thin foils to identify nanoscale precipitates and dislocation configurations.

## 3. Results

### 3.1. Corrosion Kinetics

**Figure 2:**
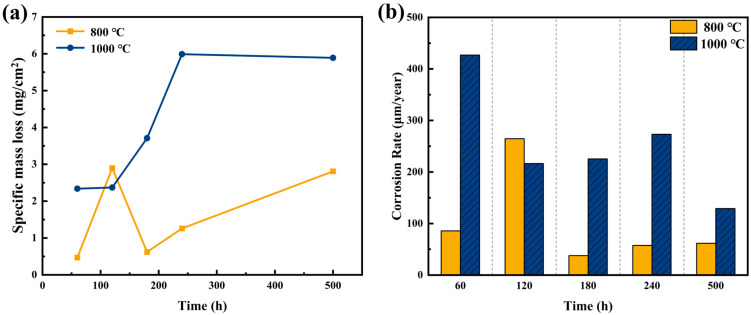
Corrosion kinetics of AFA alloys in molten carbonate at 700 °C: (**a**) time-dependent mass loss per unit area; (**b**) evolution of corrosion rate over 500 h exposure.

The corrosion resistance of the AFA alloy in molten Li–Na–K carbonate at 700 °C was evaluated by mass loss measurements. Figure 2a presents the specific mass loss (Δm/S_0_) as a function of exposure time. The specimen annealed at 800 °C exhibits significantly superior corrosion resistance compared with its 1000 °C counterpart. While both alloys show limited degradation in the initial transient stage (<60 h), the 1000 °C specimen undergoes accelerated mass loss with prolonged exposure, indicative of breakaway oxidation behavior.

The evolution of the corrosion rate is shown in Figure 2b. For the 800 °C specimen, the rate initially increases to a peak of about 265 μm/yr, but subsequently drops and stabilizes at a low level (~62 μm/yr after 500 h). This apparent self-healing behavior suggests the successful establishment of a protective passivation layer. The mass loss data for the 800 °C specimen can be reasonably fitted by a parabolic trend, which indicates that the corrosion process is diffusion-controlled and governed by solid-state transport of ions through a growing, dense oxide scale [23]. In stark contrast, the 1000 °C specimen maintains a significantly higher corrosion rate throughout the test and deviates from parabolic kinetics toward nearly linear behavior, indicative of a non-protective or mechanically unstable scale. Each data point in Figure 2 represents the average of three parallel specimens.

### 3.2. Phase Identification of Corrosion Products

**Figure 3:**
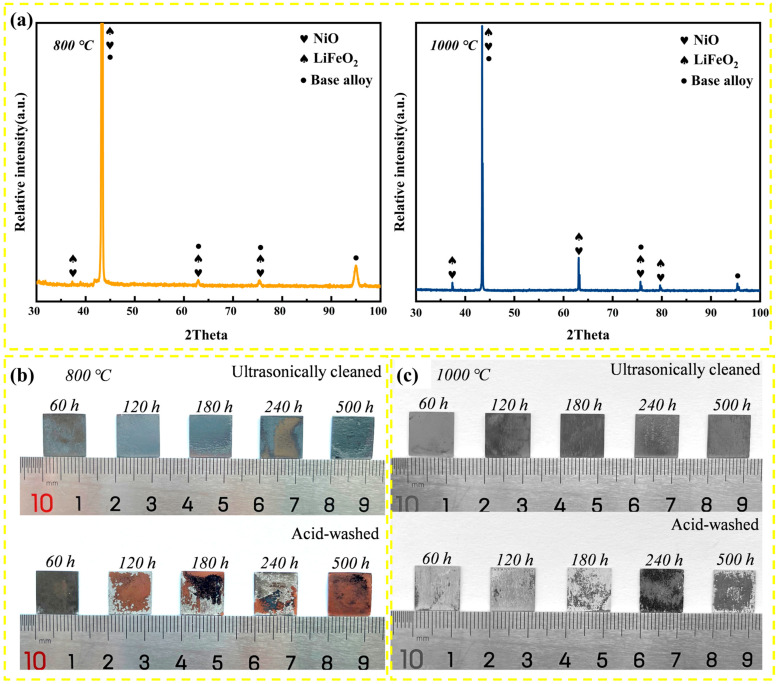
Phase and macroscopic characterization of the corroded specimens: (**a**) XRD patterns of the surface scales after 500 h; (**b**,**c**) macroscopic surface morphologies of the 800 °C and 1000 °C-annealed specimens, respectively, before and after descaling.

Figure 3a displays the XRD patterns of the corroded surfaces after 500 h. Both specimens exhibit diffraction peaks characteristic of LiFeO_2_ and NiO. It is noteworthy that the lattice parameter of non-stoichiometric Li_x_Fe_1−x_O (rock-salt structure) is very close to that of the austenitic matrix (a_matrix_ ≈ 3.59 Å). The slight broadening and superposition of the peaks around 43° suggest a contribution from both the (200) reflection of the matrix and the (200) reflection of the oxide solid solution. The absence of distinct complex spinel peaks implies that, in this highly basic melt, simple oxides and lithiated oxides are thermodynamically favored. This phenomenon of peak overlap between NiO/LiFeO_2_ and the substrate has also been reported in Ni–Fe alloys exposed to molten carbonates [24]. The strong substrate reflections further indicate that the oxide scale is relatively thin or porous, allowing X-ray penetration. No distinct diffraction peaks for Al-based oxides were detected, likely due to their low volume fraction or subsurface location, necessitating further cross-sectional analysis.

### 3.3. Surface Morphology Evolution

The macroscopic appearance of the corroded specimens varies distinctively with annealing history. Figure 3b,c compare the surface morphologies before and after the descaling process. For the 800 °C specimen (Figure 3b), the oxide scale appears compact and uniform. Even after standard acid cleaning, the underlying metal surface remains relatively smooth, confirming minimal localized attack and uniform corrosion. Conversely, the 1000 °C specimen (Figure 3c) develops a thick, rough, and loosely adherent scale. After descaling, the substrate reveals severe roughness and deep pitting, corroborating the high mass loss observed in kinetic measurements.

**Figure 4:**
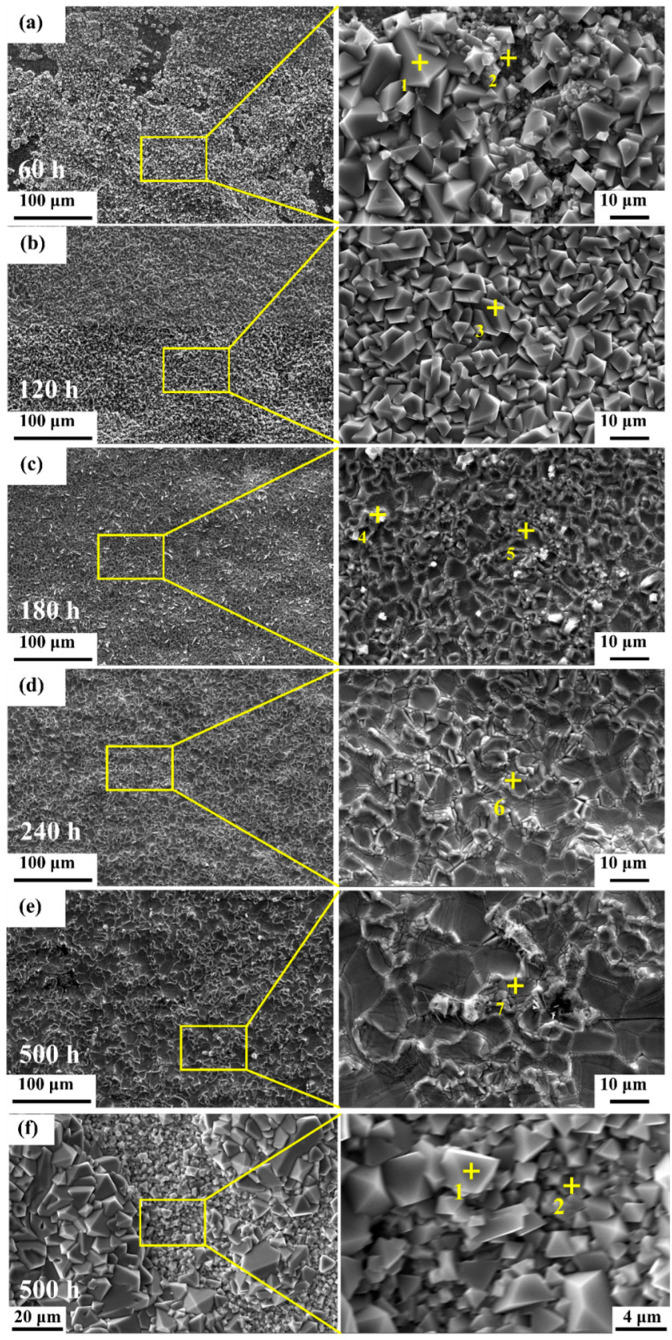
Surface morphology evolution of the oxide scales: (**a**–**e**) SEM images of the 800 °C-annealed specimen after exposure for 60 h, 120 h, 180 h, 240 h, and 500 h; (**f**) surface morphology of the 1000 °C-annealed specimen after 500 h. (The numbers 1–7 and the ”+” symbol indicate the specific locations for EDS point analysis).

Microscopic SEM examination (Figure 4a–e) of the 800 °C specimen reveals the growth mechanism of the outer scale. At 60 h, isolated spinel-like crystallites nucleate on the surface. With increasing time (120–500 h), these faceted grains coarsen and coalesce into a dense, continuous layer (Figure 4e). EDS analysis (Table 2) confirms that these outer crystals are Fe-rich oxides (consistent with LiFeO_2_). For the 1000 °C specimen (Figure 4f), the surface oxide morphology is more heterogeneous, featuring large polyhedral grains mixed with clusters of fine particles. EDS analysis (Table 3) indicates a similar Fe-rich composition, but the porous nature of this scale implies it offers little protection against melt penetration. For the 1000 °C specimen, severe internal oxidation and breakaway scale growth occurred rapidly during the early stages of exposure, leading to a highly chaotic surface evolution. Therefore, the morphology after 500 h (Figure 4f) is presented to represent its ultimate non-protective state, which features large polyhedral grains mixed with clusters of fine particles.

### 3.4. Cross-Sectional Microstructure and Element Distribution

**Figure 5:**
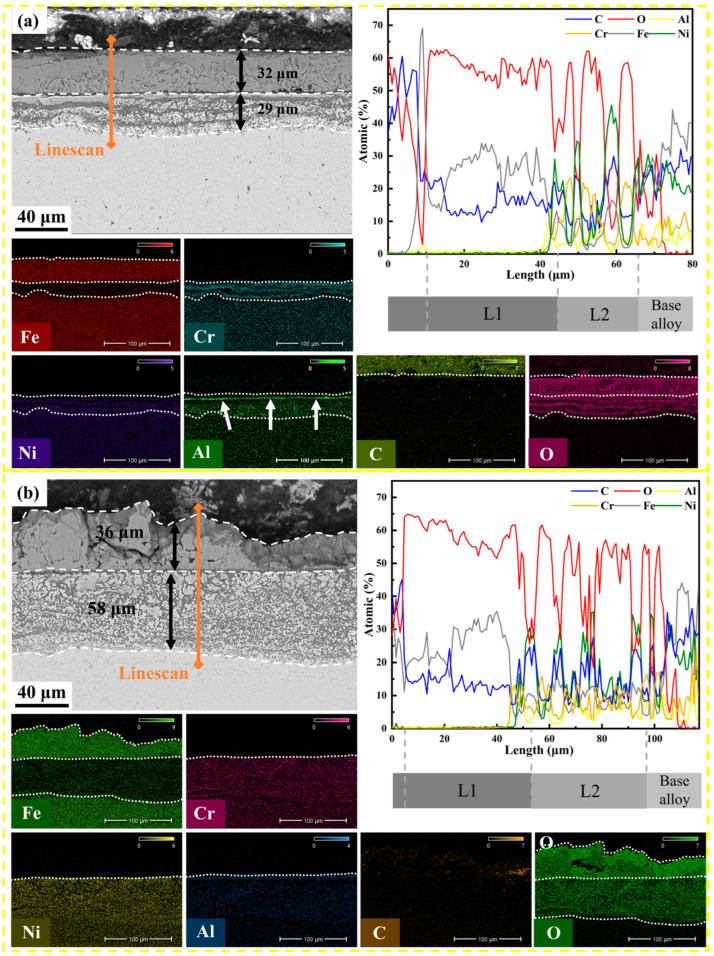
Comparative cross-sectional analysis of the oxide scales after 500 h exposure: (**a**) 800 °C-annealed specimen, showing a thin, protective scale with a continuous Al-rich inner band (confirmed by EDS maps and line scan); (**b**) 1000 °C-annealed specimen, showing a thick, non-protective scale with deep internal oxidation and absence of Al enrichment at the interface. The white arrows specifically point to the areas of Al diffusion, highlighting the Al-rich protective layer. The white dashed lines delineate the elemental diffusion zones across the interface.

To elucidate the protective mechanism, cross-sectional analyses were performed. Figure 5a reveals the corrosion architecture of the 800 °C specimen after 500 h. The total affected depth is approximately 32 μm thick. A detailed inspection of the element line scans reveals a distinct multi-layered structure. The outermost layer is confirmed to be Fe-rich LiFeO_2_, which is porous and non-protective. Beneath this Fe-rich zone, a local enrichment of Ni is observed, likely due to the selective dissolution of Fe and Cr into the melt, leaving a noble Ni-rich metallic sub-layer. Most importantly, crucial evidence of protection is found at the scale/metal interface: EDS mapping clearly identifies a continuous Al-rich band beneath the outer iron oxides. The sharp overlap of Al and O signals strongly suggests the presence of a continuous alumina (Al_2_O_3_) and/or lithium aluminate (LiAlO_2_) film. This inner layer acts as a robust diffusion barrier, effectively hindering the inward transport of oxygen and carbon.

In comparison, the 1000 °C specimen (Figure 5b) exhibits a much thicker degradation zone (~58 μm), representing an ~80% increase in depth compared to the 800 °C case. More critically, no continuous Al-rich layer is observed at the interface. Instead, the Al signal is diffuse or absent, indicating that the coarse-grained microstructure failed to supply sufficient Al flux to sustain a protective alumina-based scale. Consequently, the molten salt penetrated deeper, leading to severe internal oxidation and higher corrosion rates.

## 4. Discussion

### 4.1. Microstructural Evolution Induced by Thermomechanical Treatment

**Figure 6:**
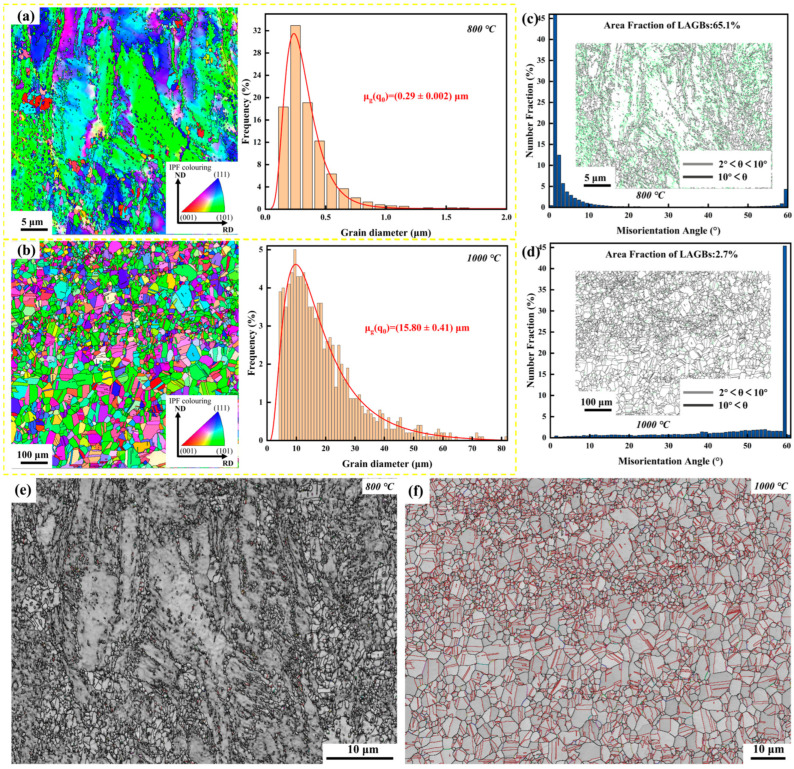
Microstructural characterization by EBSD: (**a**) 800 °C and (**b**) 1000 °C inverse pole figure (IPF) maps; (**c**) 800 °C and (**d**) 1000 °C statistical distribution; (**e**) 800 °C and (**f**) 1000 °C distribution maps of Coincidence Site Lattice (CSL) boundaries.

The thermomechanical history profoundly tailored the alloy microstructure, creating two distinct regimes of grain boundary characteristics and defect densities. As revealed by the EBSD maps in Figure 6, the 800 °C specimen retains a partially recrystallized microstructure with ultrafine grains (~0.29 μm) and a strong (101)/(111) texture. This texture evolution is typical for cold-rolled fcc alloys, where the stored energy of deformation drives the early stages of recovery but is insufficient to trigger complete grain growth at 800 °C. In contrast, the 1000 °C annealing led to full recrystallization and significant grain growth (~15.8 μm), eliminating the deformation texture and creating a stress-free lattice.

**Figure 7:**
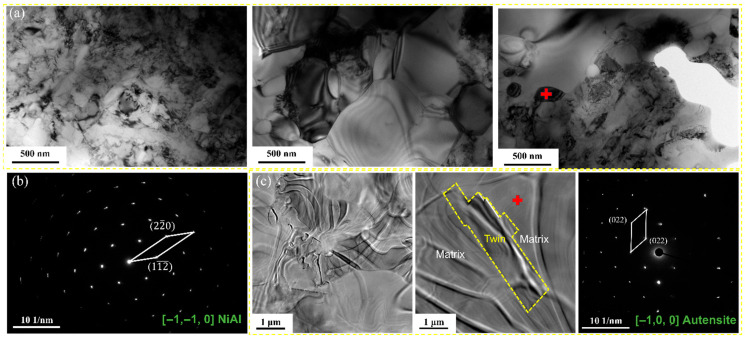
TEM analysis of dislocation configurations and precipitates: (**a**) bright-field image of the 800 °C specimen showing high dislocation density; (**b**) SAED pattern confirming the presence of NiAl precipitates in the 800 °C specimen; (**c**) bright-field image of the 1000 °C specimen showing a dislocation-free recrystallized matrix. The “+” symbols in (**a**,**c**) indicate the specific locations for TEM point analysis.

The difference in precipitate behavior is equally striking and physically significant. TEM analysis (Figure 7a,b) confirms that the high dislocation density in the 800 °C specimen accelerated the nucleation of nanoscale NiAl (B2) precipitates. Mechanistically, dislocations reduce the activation energy barrier for nucleation by accommodating the lattice misfit strain between the bcc-NiAl precipitates and the fcc-austenite matrix [25]. The dislocation cores act as preferred nucleation sites, leading to a fine dispersion of precipitates. Conversely, the 1000 °C specimen (Figure 7c) exhibits a dislocation-free matrix with negligible NiAl precipitation (i.e., an almost complete absence of the phase, as the high temperature causes complete dissolution of Al and Ni, and the lack of defects suppresses reprecipitation during cooling). At this elevated temperature, the solubility of Al and Ni in the austenite increases, favoring a solid solution state. Upon cooling, the lack of heterogeneous nucleation sites suppresses reprecipitation, leaving the Al atoms trapped in the lattice solid solution where their mobility is limited.

Furthermore, the distribution of these NiAl precipitates plays a strategic role. Being coherent or semi-coherent with the matrix, they can readily dissolve upon local Al depletion to replenish the matrix Al content [26]. In the 800 °C specimen, the dispersion of fine NiAl particles along dislocation lines ensures a uniform “local supply” of Al directly to the fast-diffusion channels [27,28]. This synergistic coupling—reservoirs (precipitates) connected to highways (dislocations)—creates a highly efficient supply chain for sustaining the scale growth, a mechanism absent in the coarse-grained 1000 °C specimen [29,30].

### 4.2. The Decisive Role of Defects on Al Diffusion Kinetics

The superior corrosion resistance of the 800 °C specimen presents an apparent paradox when viewed through classical grain boundary engineering (GBE) theory. Typically, a high fraction of low-energy CSL boundaries is desired for corrosion resistance because they resist solute segregation and intergranular attack [31]. The 1000 °C specimen indeed possesses a high fraction (46.8%) of CSL boundaries (Figure 6e,f). However, this classical view applies primarily to intergranular corrosion resistance at relatively low temperatures, where the goal is to prevent grain boundaries from being preferentially attacked. In the context of alumina-forming alloys in aggressive high-temperature environments, the priority shifts to solute supply. CSL boundaries, due to their high atomic matching and low free volume, are less effective diffusion paths compared with random high-angle boundaries [32]. Consequently, the effective Al flux in the 1000 °C specimen was insufficient to sustain an external alumina-based scale, leading to internal oxidation.

In contrast, the 800 °C specimen is dominated by low-angle grain boundaries (LAGBs, 65.1%) and a high density of geometrically necessary dislocations (Figure 6c,d and Figure 7a). These LAGBs and associated dislocation networks act as a dense array of short-circuit diffusion paths. The kinetic benefit of this microstructure can be understood via the concept of effective diffusivity (D_eff_). According to the Hart model, at the intermediate temperature of 700 °C (which is approx. 0.5–0.6 T_m_ of the alloy), lattice diffusion is sluggish [33], and total transport is dominated by short-circuit paths [34]:

D_eff_ = (1 − F_GB_ − f_pipe_) D_lattice_ + f_GB_ D_GB_ + f_pipe_ D_pipe_ (2)

where f_GB_ and f_pipe_ represent the volume fractions of grain-boundary and dislocation-pipe diffusion paths, respectively. The 800 °C treatment maximizes both f_GB_ (via grain refinement) and f_pipe_ (via retained dislocations).

This mechanism can be quantified using Wagner’s oxidation theory. The critical solute concentration ($N_{\mathrm{Al}}^{\mathrm{crit}}$) required to transition from internal to external oxidation is inversely proportional to the square root of the alloy interdiffusion coefficient (D_Al_) [23,35]:

(3) $$N_{\mathrm{Al}}^{\mathrm{crit}}={\left(\frac{\pi g\;\times \;V_{m}\;N_{O}D_{O}}{2D_{\mathrm{Al}}}\right)}^{1/2}$$

where N_O_D_O_ is the oxygen permeability and V_m_ is the molar volume. The 800 °C treatment, by introducing a dense network of dislocation pipes and subgrain boundaries, can substantially increase the effective D_Al_ compared with the lattice-diffusion-controlled 1000 °C specimen. Mathematically, an increase in D_Al_ in the denominator of Equation (3) lowers the critical $N_{\mathrm{Al}}^{\mathrm{crit}}$ required for protective scale formation. This explains why the 800 °C specimen could form a continuous alumina-based inner layer even though the bulk Al content (3.33 wt.%) is marginal for conventional austenitic alloys. Thus, for the present AFA alloy in molten carbonates, kinetic acceleration via defect engineering outweighs the benefits of a high CSL boundary fraction from a corrosion-resistance standpoint.

### 4.3. Thermodynamic Analysis of Corrosion Reactions

The corrosion degradation in molten Li–Na–K carbonate is governed by a complex interplay of oxidation and lithiation reactions. From a thermodynamic perspective, the stability of oxides is dictated by the local oxygen activity (a_O2_) a(O_2_) and the basicity of the melt a(O^2−^).

Aluminum: According to the Ellingham diagram, Al has the highest affinity for oxygen. In Li-containing melts, the formation of lithium aluminate (γ − LiAlO_2_) is thermodynamically favored over pure alumina due to the highly negative Gibbs free energy of the reaction [36,37]:

Al_2_O_3_ + Li_2_O → 2LiAlO_2_ (4)

This phase is chemically very stable and only sparingly soluble in the melt, forming the primary barrier layer observed in the 800 °C specimen.

Iron and Nickel: When the Al supply is exhausted (as in the 1000 °C specimen), Fe and Ni oxidation becomes dominant. While Ni tends to form NiO or solid solutions, Iron undergoes a stepwise oxidation to Fe_2_O_3_, which further reacts with the lithium-rich melt at the interface to form lithium ferrite:

Li_2_O + Fe_2_O_3_ → 2LiFeO_2_ (5)

The formation of LiFeO_2_ explains the Fe-rich outer layer detected in XRD. However, unlike LiAlO_2_, the LiFeO_2_ layer is porous and allows electrolyte infiltration. This porosity can be rationalized by the Pilling-Bedworth Ratio (PBR). While the PBR for Al → Al_2_O_3_ is ideally ~1.28, favoring a compressive and compact scale, the complex lithiation of iron oxides results in volume expansion and internal stress generation. The formed LiFeO_2_ scale lacks the structural coherence to accommodate these growth stresses, leading to micro-cracking and detachment [38]. This mechanical instability creates fast-track channels for the molten salt to bypass the scale and attack the substrate directly.

Chromium (The cause of mass loss): A critical degradation mechanism in carbonate melts is the basic fluxing of Chromium. While Cr initially forms a protective Cr_2_O_3_ scale, this oxide is unstable in highly basic melts (high $a_{O^{2\text{−}}}$) [15]. It dissolves via the formation of soluble chromate ions [7]:

2Cr_2_O_3_ + 4O^2−^ + 3O_2_ → 4CrO_4_^2−^ (6)

This dissolution reaction is responsible for the transition from mass gain to mass loss observed in the later stages of corrosion kinetics (Figure 2). It highlights why conventional Cr-forming steels fail in this environment and underscores the necessity of the Al-based protection strategy proposed in this work.

### 4.4. Failure Model and Kinetic Implications

Based on the microstructural evidence, a competitive failure model is proposed.

For the 1000 °C-annealed specimen, the coarse-grained microstructure with a high fraction of CSL boundaries creates a “diffusion-starved” condition. The outward flux of Al is too slow to cover the surface before Fe and Ni oxidize. Consequently, a mixed non-protective scale (LiFeO_2_ + NiO) forms rapidly. The molten salt penetrates through the pores and cracks of this scale, leading to deep internal oxidation and continuous consumption of the substrate, a mechanism consistent with the classical transition from external to internal oxidation described by Wagner [39].

For the 800 °C-annealed specimen, the TMT-induced defect network enables a diffusion-dominant condition. The rapid supply of Al stabilizes the surface in a LiAlO_2_-rich state before significant Fe/Cr dissolution can occur. This kinetic dominance effectively suppresses the basic fluxing of Cr (Equation (6)) by physically separating the Cr-rich substrate from the basic melt. The present study therefore demonstrates that, for AFA alloys in molten carbonates, achieving a critical defect density via TMT is as vital as alloy chemistry itself for high-temperature durability [40].

### 4.5. Long-Term Stability and Limitation Analysis

While the 500 h exposure demonstrates the superior performance of the 800 °C TMT specimen, extrapolating these results to the 30-year lifespan required for CSP plants warrants a cautious discussion of long-term stability. A primary concern for AFA alloys in service is breakaway oxidation, which occurs when the subsurface Al reservoir is depleted below a critical level [41].

In high-temperature oxidation, the sustainability of the protective scale relies on the continuous diffusion of Al from the bulk alloy to the scale/metal interface. As oxidation proceeds, an Al-depletion zone inevitably develops beneath the scale. In the 1000 °C specimen, slow lattice diffusion cannot replenish the surface Al consumption fast enough, causing the Al concentration at the interface to drop below the thermodynamic limit required for alumina stability. This triggers the nucleation of non-protective Fe/Ni oxides, marking the onset of breakaway oxidation. In contrast, the 800 °C specimen, with its dense dislocation network, can maintain a higher Al flux, thereby delaying the development of such a critical depletion zone and extending the effective service life.

Furthermore, in the TMT-processed samples, the presence of NiAl precipitates acts as an additional buffer. According to the Lifshitz–Slyozov–Wagner (LSW) coarsening theory, the long-term stability and coarsening behavior of these precipitates will influence the sustained Al supply to the scale. Mechanical integrity under thermal cycling is another potential failure mode. Although the 800 °C specimen formed a thin, adherent scale, the coefficient of thermal expansion (CTE) mismatch between the LiAlO_2_-based scale and the austenitic matrix could generate thermal stresses during diurnal start–stop cycles of a solar power plant [42]. The refined grain structure in the 800 °C specimen provides an additional benefit here: grain boundaries can act as crack arresters and help relieve thermal creep strains, potentially offering better resistance to spallation compared with the coarse-grained 1000 °C microstructure. Future work involving cyclic thermal testing and longer-term exposures is recommended to further validate this mechanical and chemical durability. Therefore, the present 500 h data should be regarded as a mechanistic basis rather than a direct prediction of component lifetime. From an industrial perspective, the 800 °C annealing falls within standard warm-working or stress-relief temperature ranges for large-scale structural components, such as CSP heat exchangers and piping. This indicates that the proposed TMT strategy can be readily integrated into existing manufacturing lines without necessitating complex, non-standard equipment, paving the way for cost-effective scaling.

## 5. Conclusions

This work systematically investigated the corrosion behavior of an alumina-forming austenitic (AFA) alloy in molten Li–Na–K carbonate at 700 °C, elucidating the critical interplay between thermomechanical processing, microstructural defects, and oxidation kinetics. The main conclusions are as follows:

1. Efficacy of defect engineering: A microstructure-oriented TMT strategy was successfully demonstrated. The specimen annealed at 800 °C exhibited superior corrosion resistance, with a low steady-state corrosion rate of ~62 μm/yr, significantly outperforming the coarse-grained 1000 °C counterpart. This confirms that TMT is a viable pathway to enhance the applicability of AFA alloys in aggressive molten salts without relying on expensive alloying additions.
2. Mechanism of kinetic acceleration: The superior performance of the 800 °C specimen is governed by short-circuit diffusion. While classical GBE theory favors low-energy CSL boundaries for corrosion resistance, this study reveals that, for diffusion-limited AFA systems in molten carbonates, a network of dislocation pipes and subgrain boundaries increases the effective diffusivity of Al. This kinetic advantage lowers the critical solute concentration $N_{\mathrm{Al}}^{\mathrm{crit}}$ required for passivation, enabling the rapid establishment of a protective scale even with a marginal bulk Al content.
3. Thermodynamic and mechanical stability of scales: The accelerated Al supply in the 800 °C specimen promotes the formation of a continuous, thermodynamically stable LiAlO_2_-rich inner layer. This layer acts as a highly protective barrier that effectively suppresses the basic fluxing of chromium (${CrO}_{4}^{2-}$ formation). In contrast, the 1000 °C specimen forms a porous LiFeO_2_/NiO scale, which fails due to high PBR-induced stresses and internal stress accumulation, leading to breakaway oxidation.
4. Long-term stability strategy: The study identifies a strategic trade-off: while enhanced diffusion accelerates scale formation, it also risks faster depletion of the Al reservoir. However, in the 800 °C specimen, dispersed NiAl precipitates act as localized Al buffers, mitigating the risk of premature Al depletion. This defect-assisted passivation strategy provides a metallurgical guideline for designing durable AFA alloys for next-generation CSP applications operating in high-temperature molten salts.

## Figures and Tables

**Table 1:** Chemical composition of the experimental AFA alloy (wt.%).

Elements	C	Al	Cr	Ni	Ti	Fe
Content	0.028	3.33	12.49	31.41	0.1	Bal.

**Table 2:** EDS elemental analysis (at. %) of the surface of AFA alloy after 800 °C annealing and subsequent corrosion for various exposure times (corresponding to Figure 4a–e).

Location	O	Al	Fe	Ni	Cr
Point 1	58.3	1.9	36.7	2.3	0.5
Point 2	48.8	3.2	24.5	15.4	7.9
Point 3	49.1	1.4	49.1	0.4	0.1
Point 4	57.2	0.7	39.6	0.9	0.2
Point 5	47.5	1.4	46.2	4.7	0.0
Point 6	56.9	2.9	33.9	3.9	2.2
Point 7	52.7	1.7	41.6	3.8	0.0

**Table 3:** EDS elemental analysis (at. %) of the surface of AFA alloy after 1000 °C annealing and 500 h corrosion (corresponding to Figure 4f).

Location	O	Al	Fe	Ni	Cr
Point 1	50.3	1.6	42.1	2.5	2.8
Point 2	42.7	1.2	49.7	1.8	4.3

## Data Availability

The original contributions presented in the study are included in the article. Further inquiries can be directed to the corresponding author.
